# Editorial: Global surgery: the next Frontier in global public health

**DOI:** 10.3389/fpubh.2023.1293880

**Published:** 2023-10-06

**Authors:** Jaymie A. Henry, Lye-Yeng Wong, Emmanuel Ameh, Cheng Har Yip, Andrew Hill

**Affiliations:** ^1^Baylor College of Medicine, Houston, TX, United States; ^2^Stanford Healthcare, Stanford, CA, United States; ^3^National Hospital, Abuja, Nigeria; ^4^Subang Jaya Medical Centre, Subang Jaya, Selangor Darul Ehsan, Malaysia; ^5^The University of Auckland, Auckland, New Zealand

**Keywords:** global surgery, global surgery collaboration, global public health, surgical safety checklist, trauma surgery

The delivery of surgical care globally represents the challenging nexus of colliding worlds in healthcare—public health, public finance, education, innovation, policy, and technology. Significant gains in controlling infectious diseases have been the banner of success of the public health world; but ecological changes with globalization, overpopulation, demographic shifts of an aging population facing an increasingly industrialized society, and more recently, the devastating effects of climate change and pandemics have awakened the community on the need to address the oft neglected issues such as health systems strengthening at the local and national level. Global surgery, in particular, has surfaced as a distinct area of work in the last 8 years. But what is Global Surgery? How and who has defined its limits?

It was with these questions that we launched this series, issuing a call for substantive work not only to reflect current paradigms and models on the ground but also to practically guide any stakeholder in designing further systems of change one wishes to undertake. And we were not disappointed. From this Research Topic we see a transition from an era of describing the scope and magnitude of need, to now piloting and implementing new systemic changes to increase quality and access to safe surgery. As we have called for systems change efforts, we now present findings in three major areas of work that we have identified previously (Henry et al.): addressing surgical site infections, including antimicrobial resistance (Khalid et al.; Naylor et al.; Liu et al.), maternal health (Sufian et al.; Negesse and Abebe; Dohmen et al.; Adugna et al.), and trauma care (Heris et al.; Du et al.). We also received insight into the development of subspecialty services such as pediatric surgery in Malawi (Monaghan et al.), and the impact of COVID on a pediatric cardiac service in South Africa (Aldersley et al.). Lastly, we present strong messages from important stakeholders that no change can happen without local leaders driving the agenda at the district level (Pittalis et al.) and showcase the largest regional action plan involving 32 countries in Sub-Saharan Africa dedicated toward strengthening surgical, obstetric, trauma, and anesthesia care in the region, driven by local leaders (M'pele et al.).

Albeit representing only a minute fraction of the breadth of activity taking place in the world of global surgical care delivery, this Research Topic provides valuable insights on the complexity of change efforts and the culturally sensitive nuances that are necessary to understand how it takes place at the grassroots level. Moreover, it provides broad overviews of relevant issues and the gaps in literature and practice that we need to address. It also underscores several realities: that not only are there severe deficits in infrastructure and workforce capabilities at the district level, but that what it takes to improve the quality and safety of providing care is a long and arduous process of relationship-building, teamwork, and empowerment. Indeed, Pittalis et al. demonstrated that local capacity building is the key driver to addressing barriers to surgical care at the district level in Malawi, Zambia, and Tanzania. Stakeholders in this study expressed that more training, feedback, and supervision from referral centers would help non-specialized providers practice at the highest of their capability, which would subsequently relieve bottlenecks and long patient wait times at urban tertiary care centers. Although the World Health Organization (WHO) surgical safety checklist has been shown to decrease morbidity and mortality in several well-designed studies (1), widespread adoption still remain a challenge. The mixed-methods study by Khalid et al. used a behaviorally anchored rating scale to evaluate checklist use in operating rooms in Pakistan and concluded that multi-disciplinary training is effective in improving checklist adherence across healthcare worker types. Establishment of antimicrobial stewardship (AMS) programs is another effective tool to prevent injudicious use of antibiotics and development of antimicrobial resistance, key issues in surgical care management (1). Interestingly, although Liu et al. showed that publications on AMS have risen significantly over the last few decades, most of these investigations remain centered in high-income countries. For example, Naylor et al. developed a sophisticated decision tree to estimate the burden of AMR on surgical patients using national data from England. While this tool will be helpful for other settings that are refining national surgical plans, there is still a need to contextualize the data into resource-limited settings, which stresses the importance of producing generalizable and adaptable frameworks in global surgery.

This Research Topic also addresses one of the most pressing surgical public health issues, especially in LMICs: trauma care. Evidence has shown that both pre-hospital and in-hospital capacities can significantly decrease trauma morbidity and mortality with figures noted from 25 to 47%, (2, 3) more impactful in rural communities. Heris et al. reviewed existing evidence regarding trauma-informed public health emergency responses for First Nations, or indigenous, communities, identifying the six pillars of a trauma-informed and culturally—responsive public health emergency framework that should be integrated into the provision of all emergency care. The management of major trauma has also changed globally and Du et al. demonstrated emerging trends in the management of traumatic brain injury, massive hemorrhage, and neurocritical care which highlights current research hotspots on a global scale. Lastly, best practices in maternal and perinatal health have been equally emphasized. Our topic features three studies performed in Ethiopia that range in study interest from identifying modifiable risk factors in preterm labor, to assessing factors of respectful maternity care, and evaluating birth and complications readiness in male partners. On the other hand, Dohmen et al. discuss value-based maternity care in Kenya implemented through a digital exchange platform. The interconnection of community access and national policy will continue to be the meeting point for future innovations in global surgery.

In conclusion, we hope that this Research Topic plays a role in advancing the global roadmap for affordability, access, and availability of safe surgery in LMICs. Taking a step further, we offer themes such as integration, escalation, and maturation as key strategies for implementation, adherence, and scalability of surgical systems. As such, these systems can never exist in isolation as they represent a continuum of care that begins at the community level with health-seeking behavior, continues on with intermediaries to link the patient with the healthcare system (integration) and once in the system, ensures safe and affordable care (escalation), and reintegrates the patient back to their families and communities with appropriate support and rehabilitation as necessary (maturation). By turning recommendations into clinical practice and policy, we can achieve surgical excellence through evidence-based quality improvement initiatives. Collaborative efforts driven by local leadership will be the cornerstone to producing sustainable change in global surgery.

## Author contributions

JH: Conceptualization, Funding acquisition, Supervision, Validation, Writing—original draft, Writing—review and editing. L-YW: Conceptualization, Project administration, Supervision, Validation, Writing—original draft, Writing—review and editing. EA: Project administration, Supervision, Writing—review and editing. CY: Conceptualization, Project administration, Supervision, Writing—review and editing. AH: Conceptualization, Formal analysis, Project administration, Supervision, Validation, Writing—review and editing.
